# Ovarian Cancer Incidence in the Multi-Ethnic Asian City-State of Singapore 1968-2012

**DOI:** 10.31557/APJCP.2019.20.12.3563

**Published:** 2019

**Authors:** Jeff Yi-Fu Hwang, Wei-Yen Lim, Chuen Seng Tan, Sheow Lei Lim, John Chia, Khuan Yew Chow, Wen Yee Chay

**Affiliations:** 1 *Saw Swee Hock School of Public Health, National University of Singapore. 12 Science Drive 2, #10-01, *; 2 *Tan Tock Seng Hospital, 11 Jalan Tan Tock Seng, *; 3 *KK Women's and Children's Hospital. 100 Bukit Timah Road,*; 4 *National Cancer Centre Singapore. 11 Hospital Drive, *; 5 *National Registry of Diseases Office, Health Promotion Board. 3 Second Hospital Avenue, Singapore. *

**Keywords:** Ovarian carcinoma, epidemiology, period effect, Singapore, reproductive factors

## Abstract

**Purpose:**

We investigate ovarian cancer incidence between 1968 and 2012 in Singapore, a multiethnic Asian city state.

**Methods:**

Aggregated data of ovarian epithelial cancer numbers and estimated person-years from 1968 to 2012 were obtained from Singapore Cancer Registry. Age-Period-Cohort modelling was performed.

**Results:**

The age-standardised incidence rate of ovarian cancer increased from 5.8 to 12.5 per 100,000 per year between 1968 and 2012, while the age-standardised mortality rate has remained stable. This increase was higher among Malays (5.1 to 14.0 per 100,000 per year), compared to Chinese and Indians. Serous carcinoma showed the greatest increase in incidence from 0.4 to 3.4 per 100,000 per year. Period effects were seen in the ovarian cancer incidence trend in Chinese women, but not Malay and Indian women. Clear cell and mucinous carcinoma subtypes were more common in Chinese than in Malay and Indian women. Stage at diagnosis for the years 2003-2010 differed by subtype, and the majority of patients with serous carcinomas presented at a later stage compared to those with clear cell or mucinous carcinomas.

**Conclusion:**

Ovarian cancer incidence rates have doubled in 40 years in Singapore. There were ethnic differences in incidence rates and ovarian cancer subtypes.

## Introduction

Ovarian cancer is a serious disease, and close to 70% of patients who are diagnosed at stage III and IV succumb to it (Dinkelspiel et al., 2015). Internationally, it is the 7th most common cancer among women, contributing 2.4% of all incident cases of cancer among women, and accounting for 4.4% of all cancer deaths (International Agency for Research in Cancer [IARC], 2012). In many countries across Asia, the incidence of ovarian cancer has been rising (Lee et al., 2014; Huang et al., 2016). In Singapore, ovarian cancer is the 5th most common female cancer, constituting 5.4% of new cancer cases and 5.1% of all female cancer deaths. The majority of ovarian cancers are diagnosed at an advanced stage (Maringe et al., 2012), but screening strategies for ovarian cancer in a low-risk population have thus far not been shown to be effective (Jacobs et al., 2015; Kobayashi et al., 2008; Buys et al., 2011; Henderson et al., 2018).

Reproductive factors play a strong role in the pathogenesis of ovarian cancer. High parity has been noted to be a strong protective factor (Schuler et al., 2013), whilst endometriosis (often associated with nulliparity) is associated with clear cell and endometrioid ovarian cancer (Hunn and Rodriguez, 2012; Choi et al., 2007). Germline mutations such as BRCA1 and BRCA2 are strong risk factors in hereditary ovarian cancer, which account for 20% of all cases of ovarian cancer. Conversely, oral contraceptive pills have been found to be protective - both against sporadic and BRCA-associated ovarian cancers. Smoking and obesity are also suspected to be minor adverse risk factor (Hunn and Rodriguez, 2012). Overall, the evidence suggest that reproductive hormone exposure plays an important role in the pathogenesis of ovarian cancer. 

Singapore is a multi-ethnic city-state at the tip of the Malay peninsula. The resident population of Singapore has grown from 2.01 million in 1968 to 3.82 million in 2012. Ethnic composition in Singapore is about 75% Chinese, 14% Malay, 8% Indian and 3% other ethnic groups, and this ethnic distribution has remained largely similar over the last 40 years. Many Asian countries, especially in East Asia and including Singapore, have experienced an extreme demographic transition as women delay childbearing and have fewer children, resulting in declining fertility rates (World Bank, 2018). There have been several studies that have looked at secular trends in ovarian cancer incidence rates in Asia in the context of substantial socio-economic changes. Almost all studies have shown an increase in incidence over the last 2-3 decades. In this study, we investigate ovarian cancer incidence between 1968 and 2012 in Singapore, explore ethnic differences in incidence rates and examine age, period and cohort effects using Age-Period-Cohort (APC) analysis. 

## Materials and Methods

The Singapore Cancer Registry (SCR) has collected data on all incident cases of ovarian cancer in Singapore since 1968. Multiple sources are used, including notifications by the medical profession, pathology records, hospital records, and mortality data from the Registry of Births and Deaths. Death reporting, including reporting of cause of death is mandatory in Singapore. All incident cases of epithelial ovarian cancers that were diagnosed among Singapore citizens and permanent residents in the period January 1968 to December 2012 were included in the study. Ovarian cancer subtype data were available to December 2010. Teratomas, immature teratomas, germ cell tumours, borderline tumours, and sex cord stromal tumours were excluded. Cancer classification was done using the International Classification of Diseases, 9th edition and the Manual of Tumour Nomenclature and Coding (MOTNAC) up to 1992. The International Classification of Diseases for Oncology, 2nd edition was used from 1993 to 2002, and the International Classification of Diseases for Oncology, 3rd Edition (ICD-O-3) was used thereafter (National Registry of Diseases Office Singapore [NRDO], 2015). Population estimates of Singapore citizens and permanent residents maintained by the Singapore Cancer Registry that were generated through intra- and extrapolation of population figures from the decadal censuses of 1970, 1980, 1990 and 2000 (NRDO, 2010) were used for the period 1968-2007, while mid-year population estimates from the Department of Statistics Singapore were used for the period 2008-2012. Segi’s World Population was used in direct standardisation to calculate age-standardised rates (Ahmad, 2001). Fertility data of Singapore was obtained from Population Trends 2012 (Department of Statistics Singapore, 2012), an annual publication by Singapore Department of Statistics, and a study by Saw, which estimated ethnic-specific total fertility rates annually before 1987 (Saw, 1990). 

Age-Period-Cohort (APC) modeling (Holford, 1983) was performed to assess the relative importance of age, period and cohort effects in incidence risk. The following models were considered: a model with age alone, an age-drift model where the drift term was the linear temporal variation of rates indistinguishable as either period or cohort influences, age-period and age-cohort models, and a full APC model. A model-building approach was used, using Likelihood Ratio test (LR test) and the Akaike Information Criterion (AIC) to determine the most parsimonious model that fits the data. The goodness of fit of the models was determined using the deviance statistic. The best-fit model was selected by first checking the deviance statistics for good fit (p>0.05). Then the best-fit model was selected among those models with good fit on the basis of the lowest AIC value, and a LR test ratio p<0.05 for nested comparisons with the previous less complex model. The exact linear relationship between age, birth cohort and period of cancer diagnosis results in an identifiability problem that does not allow simultaneous estimation of all three effects. To circumvent this problem, we set the lowest 2 age-groups as identical in the full model. This is based on the understanding that cancer rates in the 20 to 29-year age group are largely similar, without a large increase in risk over that 10-year age band. All analyses were performed using STATA version 14 (StataCorp, 2015) and R version 2.14.0 (R Core Team, 2013). This study used aggregated data that is available in the public domain, without individual-level data, and as such, ethics clearance is not required. 

## Results

**Figure 1:**
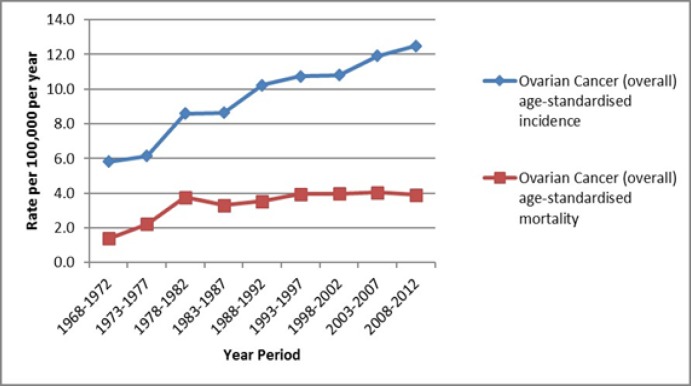
Age-Standardised Incidence and Mortality Rates of Ovarian Cancer in Singapore 1968-2012

Overall, age-standardised incidence of ovarian cancer has increased from 1968 to 2010, from 5.8 per 100,000 per year in the period 1968-1972, to 12.5 per 100,000 per year in 2008-2012, representing a greater than doubling in incidence. In comparison, the age-standardised mortality rate of ovarian cancer has remained relatively stable. After an increase in mortality rate from 1.4 per 100,000 per year in 1968-1972, to 3.8 per 100,000 per year in 1978-1982, mortality rate has remained at 3.3 to 4.0 per 100,000 per year since then to 2012 (Figure 1).

**Figure 2:**
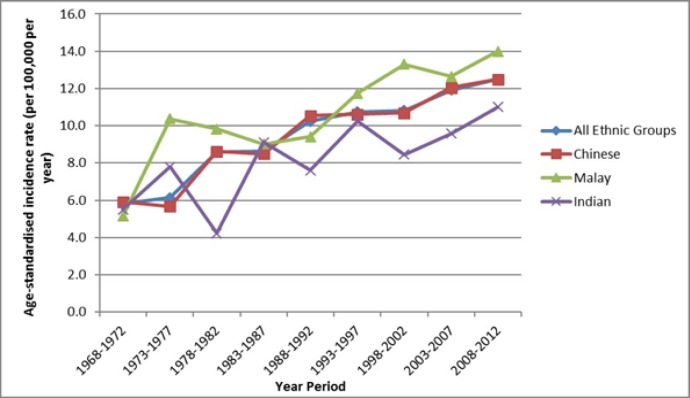
Age-Standardised Incidence of Ovarian Cancer by Ethnicity in Singapore 1968-2013

The age-standardised incidence rates of ovarian cancer for Chinese, Malays and Indians have all increased over the period under observation (1968-2012) (Figure 2). Malays have the highest incidence (14.0 per 100,000 per year in 2008-2012), followed by Chinese (12.5 per 100,000 per year) and Indians (11.0 per 100,000 per year).

**Figure 3:**
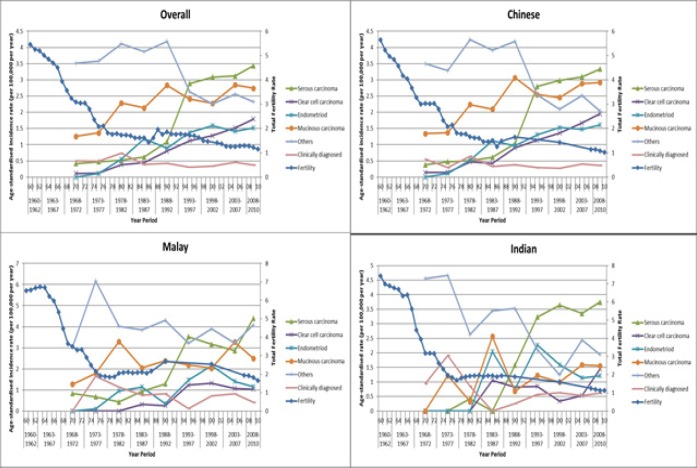
Overall Age-Standardised Incidence of Ovarian Cancer by Subtype, in All Women, Chinese Women, Malay Women and Indian Women, with Total Fertility Rates, 1960-2010

Total Fertility Rate fell sharply from 5.76/female in 1960 to 1.79 in 1978, with a gentler decline from that point to 1.29 in 2012 (Figure 3). Significant drops in fertility were seen in all 3 ethnic groups. In 2012, TFR for Malays was slightly higher at 1.69 compared to 1.18 for Chinese and 1.15 for Indians. Sharp increases in ovarian cancer incidence rates were seen from 1968 to about 1988 overall, driven largely by rates in the Chinese. Overall, there has been an increase in the incidence of all ovarian cancer subtypes evaluated (serous, clear cell, endometrioid and mucinous) over time, and a decline in “Others” and clinically diagnosed cases. For all 3 ethnic groups, serous carcinoma is the most common subtype, and also the subtype with the largest absolute increase in incidence since 1968 (overall from 0.4 to 3.4 per 100,000 per year), compared to mucinous (1.3 to 2.7 per 100,000 per year), clear cell (0.1 to 1.8 per 100,000 per year) and endometrioid (0.0 to 1.5 per 100,000 per year). “Others” was the most common subtype in 1968-1972, which has declined from 3.5 to 2.3 per 100,000 per year in the latest time period (see Figure 3). Overall, the incidence of mucinous carcinoma appears to be lower in Indians than Chinese and Malays, and the incidence of clear cell carcinoma appears lower in Malays and Indians compared to Chinese (also see supplementary Figure 1).

**Figure 4:**
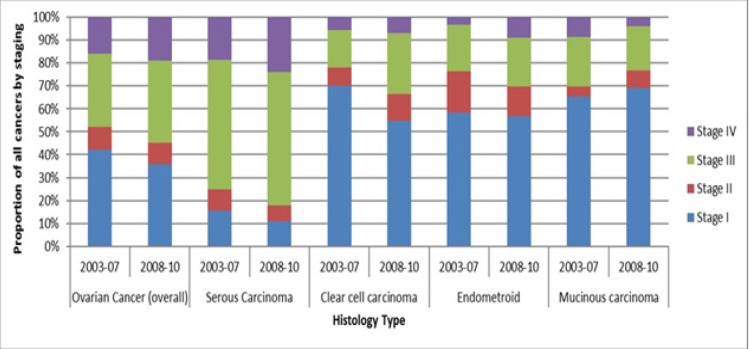
Stage of Diagnosis of Ovarian Cancer by Subtype in Singapore in Years 2003 to 2010

Overall, approximately half of all ovarian cancers in Singapore are diagnosed in Stage I and II (42% and 10% respectively in 2003-2007, and 36 % and 9% respectively in 2008-2010). Serous carcinoma, the subtype with the highest incidence, has a noticeably later stage at diagnosis (58% at stage III and 24% at stage IV in 2008-2010), compared to other subtypes of ovarian cancer (55% to 70% in stage I), see Figure 4. 

In Age-Period-Cohort modelling, based on AIC, the age-period model best describes the trend in Chinese women. This model had a good fit (p=0.20), the lowest AIC value, and had a significant p value on the Likelihood ratio test compared to the drift model, suggesting a better fit than that model. In Malay women, an age-drift model best fit the data, with good fit (p=0.67), the lowest AIC, and a significant p value when compared to the age model. In Indian women, both the age and the age-drift model had good fit. The age-drift model had a lower AIC value, but was not significantly better than the age model on the likelihood ratio test, although the p value was very close to significance (p=0.0527). Based on this data, we believe that the age-drift also best describes the temporal trend data for Indian women (Table 1).

**Figure 5:**
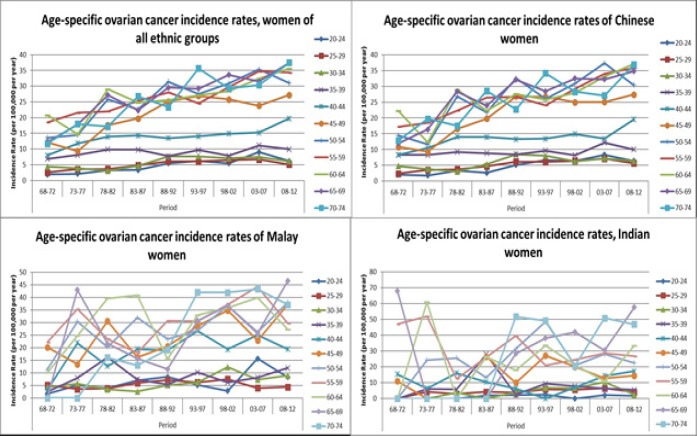
Age Specific Ovarian Cancer Incidence Rates by Period in All Women, Chinese Women, Malay Women and Indian Women, 1968-2012

Figure 5 shows the age-specific rates by periods for all women, and for women in the 3 ethnic groups. Data for women older than 74 years of age were removed because small numbers especially in the 1960s and early 1970s resulted in very unstable estimates. The graphs show rising incidence rates for almost all age groups in the Chinese from 1968-1972 to 2008-2012, supporting the period effect identified in the APC analyses. The graphs for Malay and Indian women are harder to interpret, with rates that fluctuate substantially across the 40-year period, supporting an age-drift model.

**Table 1:** Age-Period-Cohort Modelling for All Ovarian Cancers Stratified by Ethnicity 1968-2012

	Model	Deviance statistic	Degrees of freedom	Deviance P	AIC	Model-building P1
Chinese females	Age	321.59	96	<0.0001	925.1	
	Age and drift	132.11	95	0.0071	737.62	<0.00001(drift vs age alone)
	Age and period	98.92	88	0.2	718.43	<0.00001 (period vs age alone) <0.00001 (period vs drift)
	Age and cohort	113.91	77	0.004	755.42	<0.00001 (cohort vs age alone) 0.4425 (cohort vs drift)
	Age, period, cohort	84.57	70	0.1131	740.07	0.7055 (APC vs age period) <0.0001 (APC vs age cohort)
Malay females	Age	113.96	96	0.1	507.57	
	Age and drift	88.6	95	0.67	484.21	<0.00001 (drift vs age alone)
	Age and period	79.45	88	0.73	489.06	<0.00001 (period vs age alone) 0.2416 (period vs drift)
	Age and cohort	67.44	78	0.8	497.05	0.0002 (cohort vs age alone) 0.219 (cohort vs drift)
	Age, period, cohort	57.5	71	0.88	501.11	0.1869 (APC vs age period)
						0.1923 (APC vs age cohort)
Indian females	Age	108.59	96	0.18	378.43	
	Age and drift	104.84	95	0.23	376.68	0.0527 (drift vs age alone)
	Age and period	99.22	88	0.19	385.06	0.3120 (period vs age alone) 0.5851 (period vs drift)
	Age and cohort	94.58	77	0.08	402.42	0.7830 (cohort vs age alone) 0.9231 (cohort vs drift)
	Age, period, cohort	89.11	70	0.0614	410.95	0.9282 (APC vs age period) 0.6027 (APC vs age cohort)

^1^Model building: Model-building used a set of nested models: age alone, age and drift, age, drift and period, age, drift and cohort, and age, drift, period and cohort. “P values” refer to the p value for the likelihood ratio test comparing two models, one nested in the other; the models compared are in brackets. In the full APC model, the agegroup variable for the youngest agegroup was recoded to be identical to that for the next youngest agegroup.

## Discussion

Other Asian countries have reported a similar increase in incidence of ovarian cancer over time. In Hong Kong, the incidence rate of ovarian cancer has increased about 1.4% per annum from 1997 to 2006, with crude incidence of 10.8 per 100,000 women per year during the study period (Wong et al., 2012). In Taiwan, the age-adjusted incidence rate of ovarian cancer increased from 1.01 to 6.33 per 100,000 person-years from 1979 to 2008 (Chiang et al., 2013). These countries, like Singapore, have experienced dramatic declines in fertility rates. In India, the annual percentage change in age-adjusted incidence rates for ovarian cancer range from 0.19% to 4.67% in various registries, from 1982 or 1988 to 2003, with age-adjusted incidence rate for ovarian cancer about 6.0 per 100,000 (Yeole, 2008), while another study of 19 registries estimated a mean annual increase of 0.26-2.44% over about the last 2 decades (Murthy et al., 2009). In Korea, the incidence of ovarian cancer remained similar from 1999 to 2005, from 5.2 to 5.5 per 100,000 women-years (Park et al., 2010). Ovarian cancer mortality from 1979-2010 has remained stable in Hong Kong and Singapore, although it increased in South Korea (Lee et al., 2014). In Shanghai, China, over a 40-year period, ovarian cancer incidence has increased on average 1.8% per annum (Huang et al., 2016). A study in Niigata prefecture also showed increasing rates of ovarian cancers, and in particular for mucinous and clear cell adenocarcinomas (Yahata et al., 2012). Overall, ovarian cancer rates in Singapore Chinese women appear similar to rates in Hong Kong Chinese women while they are about a third higher than those in Taiwanese Chinese women. Ovarian incidence in Singapore Malay women also appear to be about a third higher than rates in Malay women in Malaysia (IARC, 2018).

Despite the increase in incidence rates, mortality rates have been relatively stable over the 40-year time period, suggesting that survival has improved. The 5-year Age-Specific Relative Survival has increased from 29.2% in the 1983-1987 period to about 45% from 1993 onwards, reported in a monograph produced by the Singapore Cancer Registry (SCR, 2015). Indeed, relative survival for ovarian cancer in Singapore was one of the highest among countries compared in the monograph, and the authors suggested that this could be due to the higher proportion of endometrioid, clear cell and mucinous subtypes of ovarian cancer in Singapore compared to other (especially Western) countries (SCR, 2015). 

Widespread screening programmes are unlikely to have contributed significantly to the improvement in survival, given that an effective ovarian cancer screening modality has not been identified, and ovarian cancer screening is not part of the national screening programme in Singapore. While increase in awareness about the disease and its symptoms could have resulted in earlier help-seeking, this is unlikely to be a main driver in survival improvement, given that the majority of serous cancers (the most common subtype in Singapore) still present at stage 3 and 4. The most likely reason for the improvement in mortality is better treatment; for example, the introduction of cis-platinum and paclitaxel as treatment corresponded in time with the decrease in five-year case-fatality rate in the U.S (Sopik et al., 2015). 

The decline in “Others” subtype likely represents improvements in histological diagnostic techniques which had resulted in classification of “NOS” cancers into an ovarian cancer subtype. There has been an increase in incidence of both clear cell carcinoma and endometrioid carcinoma, subtypes of which women with endometriosis are at higher risk (Gaducci et al., 2014; Heidemann, 2014). From 2008 to 2010, clear cell carcinoma made up 15% of the incident cases of ovarian cancer in our study population, a percentage higher than other Asian or Western countries (around 5-10%), but less than that in Japan (20-25%) (Ushijima, 2009). Asian women may have a higher prevalence of endometriosis compared to Caucasians (Gerlinger, 2012), which may explain the higher proportion of clear cell carcinoma in Singapore. 

Malay women have a higher incidence of ovarian cancer compared to Chinese and Indians, even though Malays have a higher absolute fertility rate. Malay women appear to have higher smoking rates and prevalence of obesity (Ministry of Health Singapore, 2011), factors linked to ovarian cancer. There also appears to be some subtype differences, with a lower incidence of clear cell carcinomas in Malays and Indians, and a lower incidence of endometroid carcinomas in Indians. We do not have data about the relative prevalence of endometriosis in the 3 ethnic groups in Singapore.

We notice a period effect in Chinese women, but this was not seen in Malay or Indian women. Instead, for Malay and Indian women, there were some additional effects beyond age that could not be partitioned into period or cohort effects. Our results suggest that known reproductive factors such as fertility, infertility, and oral contraceptive use do not completely explain these differences in incidence rates, and do not explain the period effect seen in Chinese women, since changes in these factors should manifest as a cohort effect rather than a period effect. The period effect could be an artefact resulting from more accurate classification of cancers as being of ovarian origin with improving medical technology, such that cancer rates appear to increase across all age groups over time. However, similar effects should also have been seen in Malay and Indian women. It is not clear what explains the period effect observed. There is evidence of substantial changes in behaviour in Singaporeans during this critical 40-year period, as it was a period of major socio-economic changes in Singapore, and it is possible that a heretofore unknown risk factor that affects mostly Chinese women across all ages might be involved.

Ethnic differences in ovarian cancer incidence and the different distribution of ovarian cancer subtypes should be explored further as these may offer clues to mechanisms of ovarian carcinogenesis. The late stage presentation of ovarian serous carcinoma is noteworthy, and suggests there is room to increase awareness amongst women of early symptoms of ovarian cancer.

A major strength of this study is the comprehensiveness of the data available, including cancer subtype information. Furthermore, differences in incidence of ovarian cancer in different Asian ethnic groups living in the same country could be explored in the setting of Singapore. This study is limited by the absence of information of the trends in major known risk factors for ovarian cancer other than fertility rate. For example, we could not assess secular trends in contraception use and tubal ligation in Singapore, although there was a very active National Family Planning Programme in the 1960s to 1980s, and a recent survey found that 20% of Singaporean females have used oral contraceptives (Gosavi, 2016). The cancer registry data might not have been complete in the years immediately after the registry was first set up. Finally, APC analysis suffers from the well-known identification problem as age, period and cohort are linked. Various statistical methods have been proposed to resolve this problem, but recent studies using different methods on the same disease trends have arrived at starkly conflicting conclusions. Harper has highlighted that designing and interpreting APC models need to be informed by substantive a priori knowledge (Harper, 2015).

In conclusion, we find an increase in ovarian cancer incidence over a 45-year period in Singapore. There were intriguing differences in ovarian cancer subtypes among the 3 major ethnic groups, and the presence of a period effect in Chinese women that was not seen in women of other ethnic groups. Finally, the relatively high proportion of late stage presentation of ovarian serous carcinoma points to a need for increasing awareness among women about early symptoms of ovarian cancer.
